# *Wenzhouxiangella* Strain AB-CW3, a Proteolytic Bacterium From Hypersaline Soda Lakes That Preys on Cells of Gram-Positive Bacteria

**DOI:** 10.3389/fmicb.2020.597686

**Published:** 2020-11-13

**Authors:** Dimitry Y. Sorokin, Damon Mosier, Jackie K. Zorz, Xiaoli Dong, Marc Strous

**Affiliations:** ^1^Winogradsky Institute of Microbiology, Federal Research Centre for Biotechnology, Russian Academy of Sciences, Moscow, Russia; ^2^Department of Biotechnology, Delft University of Technology, Delft, Netherlands; ^3^Department of Geoscience, University of Calgary, Calgary, AB, Canada

**Keywords:** lantibiotic, *Wenzhouxiangella*, denitrification, bacteriovores, proteolytic alkaline soda lake, nanopore sequencing, comparative genomics

## Abstract

A new haloalkaliphilic species of *Wenzhouxiangella*, strain AB-CW3, was isolated from a system of hypersaline alkaline soda lakes in the Kulunda Steppe using cells of *Staphylococcus aureus* as growth substrate. AB-CW3’s complete, circular genome was assembled from combined nanopore and Illumina sequencing and its proteome was determined for three different experimental conditions. AB-CW3 is an aerobic gammaproteobacterium feeding mainly on proteins and peptides. Unique among *Wenzhouxiangella*, it uses a flagellum for motility, fimbria for cell attachment and is capable of complete denitrification. AB-CW3 can use proteins derived from living or dead cells of *Staphylococcus* and other Gram-positive bacteria as the carbon and energy source. It encodes and expresses production of a novel Lantibiotic, a class of antimicrobial peptides which have so far only been found to be produced by Gram-positive bacteria. AB-CW3 likely excretes this peptide via a type I secretion system encoded upstream of the genes for production of the Lanthipeptide. Comparison of AB-CW3’s genome to 18 other *Wenzhouxiangella* genomes from marine, hypersaline, and soda lake habitats indicated one or two transitions from marine to soda lake environments followed by a transition of *W. marina* back to the oceans. Only 19 genes appear to set haloalkaliphilic *Wenzhouxiangella* apart from their neutrophilic relatives. As strain AB-CW3 is only distantly related to other members of the genus, we propose to provisionally name it “*Wenzhouxiangella alkaliphila*”.

## Introduction

Whereas pH-neutral marine and salt lake habitats mainly contain sodium chloride, a large fraction of soda lake brines consists of sodium carbonate/bicarbonate, responsible for its stable pH around 10, and molar values of alkaline buffering capacity (Jones et al., 1977; Sorokin et al., 2015; Schagerl, 2016). Bacteria thriving in soda lakes are characterized as double extremophiles, more specifically haloalkaliphiles. The haloalkaliphilic prokaryotes of soda lakes have been intensively investigated in the last several decades, both in pure cultures and by molecular ecology approaches. They are attractive for fundamental biology as double extremophiles but also present opportunities for biotechnology as producers of alkali-stable enzymes. One of the examples of the latter are alkali-stable hydrolases, including glycosyl-hydrolases and proteolytic enzymes which have important potential for application in biofuel production and laundry detergents (Horikoshi, 2006; Zhao et al., 2014; Mamo and Mattiasson, 2016; Dumorné et al., 2017). Although many alkaline serine proteases have been characterized from non-halophilic alkalitolerant and alkaliphilic bacteria, the identity of haloalkaliphilic aerobic proteolytics in soda lakes is still not well understood. So far, only three genera of dedicated proteolytics from hypersaline soda lakes have been characterized in culture. These include two members of the *Bacteroidota* – a moderately salt-tolerant “*Cyclonatronum proteinivorum*,” an extremely salt-tolerant *Natronotalea proteinilytica*, and an extremely salt-tolerant gammaproteobacterium *Natronospira proteinivora* (Sorokin et al., 2017a, c, 2018).

The genus *Wenzhouxiangella* and family *Wenzhouxiangellaceae* belong to the order *Xanthomonadales* in *Gammaproteobacteria*. So far, the genus features three validly described single-strain species, *W. marina*, *W. sediminis*, and *W. limi*, with an additional one, “*W. salilacus*,” still awaiting validation (Wang et al., 2015; Guo et al., 2016; Han et al., 2019; Zhang et al., 2020), with complete or nearly complete genomic information available for each. *Wenzhouxiangella* bacteria are aerobic, generally non-motile, and often capable of hydrolyzing proteins and/or polysacchararides. All isolates are moderately halophilic neutrophiles originating from sea water and hypersaline lakes. Furthermore, several unidentified members of this genus with sequenced genomes have been recovered from hypersaline sea solar salterns.

In recent metagenomic surveys of alkaline soda lakes in south-western Siberia and British Columbia (Vavourakis et al., 2018; Zorz et al., 2019), it appeared that members of *Wenzhouxiangella* are abundant in the soda lake environment, with relative sequence abundances >6% in some samples. However, *Wenzhouxiangella* have never been isolated from soda lakes so far.

Here, we describe the first haloalkaliphilic member of *Wenzhouxiangella*, a dedicated aerobic proteolytic bacterium isolated from a group of alkaline soda lakes in the Kulunda Steppe (Altai region, Russia). We report its phenotypic, genomic and differential proteomic properties. For this genome, we combined long-read nanopore sequencing (Jain et al., 2016) with short read Illumina sequencing to obtain a closed, complete, circular genome sequence.

By comparing nineteen nearly complete genomes of *Wenzhouxiangella* isolates, we investigate the content of core genomes of soda lake and marine/salt lake ecotypes, enabling inferences about evolutionary transitions between these two different types of saline habitats.

## Materials and Methods

### Soda Lake Samples

For four hypersaline soda lakes (Cock Soda Lake, Picturesque Lake, Bitter-1 and Tanatar-1), all located in the Kulunda Steppe, Altai region, Russia, the top 2 cm of the sediment was mixed together with near bottom brines (1:1) in 50 ml sterile Falcon tubes. The brine pH ranged from 9.7 to 10.2, the salinity from 85 to 300 g/l and soluble carbonate alkalinity – from 0.6 to 3.5 M. The samples were mixed in equal proportions and the resulted mix was used as the inoculum. The lakes were sampled in July 2015 and 2016.

### Enrichment, Isolation, Cultivation

To enrich for proteolytic and bacteriovorous bacteria, *Staphylococcus aureus* cells (strain DSM 20231) were chosen as the substrate. *S. aureus* was cultured in LB medium until early stationary growth phase, the cells were harvested by centrifugation (8,000 rpm, 20 min), the pellet was washed with tap water three times and finally the cells were resuspended in demineralized water, resulting in a suspension with OD_600_ of 20. This was autoclaved for 10 min at 120°C. The killed cells were harvested by centrifugation (8,000 rpm, 20 min) in sterile 50 ml Falcon tubes, washed two times with sterile demineralized water and resuspended to an OD_600_ of 10. The cells were then subjected to 3 min of sonication at maximum power in 5 ml portions with the sterile washing step repeated. The final preparation was kept at 4°C and added to cultures at 1:20 dilution.

The mineral base medium used for the enrichment and further cultivation of the pure culture was a sodium carbonate buffer containing 2 M total Na^+^ with a pH 9.5 and included the following (g/l): Na_2_CO_3_ 64, NaHCO_3_ 48, NaCl 18, K_2_HPO_4_ 1. The pH was adjusted to 9.5 by titration with 50% (v/v) HCl. After sterilization at 120^*o*^C for 30 min the medium was supplemented with 4 mM NH_4_Cl, 1 mM MgSO_4_ and 1 ml each of vitamin mix and trace metal solution (Pfennig and Lippert, 1966). A positive enrichment showing degradation of *Staphylococcus* cells and growth of rod-shaped cells was sub- transferred several times at 1:100 dilution in fresh medium, resulting in a sediment-free culture, which was plated on a solid medium with the same composition but containing 1 M total Na^+^ (prepared by 1:1 mixing of the base medium and 4% agar at 50°C). Finally, a pure culture was isolated from a single colony showing clarification of the opaque background around it due to partial lysis of the *Staphylococcus* cells.

### Microbial Physiology

For the salinity profile (from 0.2 to 4 M total Na^+^), two extra sodium carbonate media were prepared, containing 0.6 and 4 M total Na^+^ at pH 9.5. The media with salinity 0.2 and 0.4 M Na^+^ was obtained by dilution of the 0.6 M medium followed by pH readjustment. For higher salinity, the 0.6, 2.0, and 4.0 M bases were mixed in different proportions. For the pH profile, the media containing 2 M total Na^+^ was used together with the following buffers: for pH 7–8 (0.1 M HEPES/2 M NaCl); pH 8–8.5 (1 M NaHCO_3_/1 M NaCl); pH 9–11 (combination of NaHCO_3_ and Na_2_CO_3_). The mineral bases were filter-sterilized to avoid pH shifts and were supplemented with the same nutrient components as indicated above for the standard growth medium. The substrate for these tests was casein peptone and yeast extract (0.5 g/l each). The actual pH was measured at each sampling point. The 20 ml cultures were incubated in closed 120 ml serum bottles, placed on a rotary shaker at 150 rpm and 37°C. The growth was monitored by measuring OD_600_. In case of growth with the *Staphylococcus* cells, attempts to estimate growth by the cell protein analysis (Lowry method) failed, since the zero time controls with only *Staphylococcus* cells gave a high background values, even using mild extraction conditions, i.e., 10 min hydrolysis in 1 M NaOH instead of customary 5 min at 100°C, in hope to selectively lyse the Gram-negative cells of pure culture. Therefore, the growth was also monitored by the OD_600_ measurements and verified by microscopy. Direct microscopic cell counting was not possible, however, since the pure culture cells were growing in large aggregates.

In addition to *S. aureus*, another two Gram-positive organisms were tested as substrate for AB-CW3 – *Micrococcus luteus* (DSM 20030) and the haloalkaliphilic actinobacterium *Isoptericola* sp. DS111 (Sorokin et al., 2017b). *Isoptericola* grows as a suspension of individual cells. *Micrococcus* was grown on the same LB medium as *Staphylococcus. Isoptericola* was grown on a sodium carbonate-based medium containing 0.6 M total sodium at pH 10 with starch as substrate. The cells of both were pretreated in the same way as the cells of *Staphylococcus*.

The ability of AB-CW3 to utilize various organic compounds as growth substrate was tested in mineral carbonate-based medium at 2 M total Na^+^ and pH 9.5 containing 100 mg casein peptone as a background. The substrates were added at concentration 1 g/l. Cultivation was performed in 30 ml screw cupped flat-bottom bottles with 10 ml medium placed on a rotary shaker (150 rpm) at 37°C. Growth was monitored by OD600 in comparison to the background control. The following proteins were tested: gelatine, casein, bovine serum albumin, lactalbumin, soy protein, hemoglobin, collagen, and powdered alpha-keratin. The following sugars were tested: glucose, galactose, fructose, raffinose, mannose, rhamnose, arabinose, ribose, lactose, xylose, cellobiose, sucrose, trehalose, maltose, melezitose, melibiose, soluble starch, and dextrin. The following organic acids and lipids were tested: acetate, pyruvate, lactate, and olive oil.

The ability of pure culture to denitrify was examined in the standard growth medium containing 2 M total Na^+^ at pH 9.5, in 30 ml serum bottles. Anoxic conditions were created by three cycles of alternatingly applying a vacuum and flushing with sterile argon gas. Nitrate consumption was detected qualitatively by Merck sticks, nitrite was quantified colorimetrically (Gries-Romijn-van Eck, 1966) and N_2_O conversion to N_2_ was visualized in soft agar cultures by formation of N_2_ bubbles.

The cellular location of hydrolytic activities was tested with the qualitative agar-diffusion assay (carbonate buffer, 1.5 M total Na^+^/pH 9.5) with casein for proteolytic, starch for amylolytic and CMC for beta-1,4 endoglucanse activities, respectively (1 g/l each). The cells were first grown in the presence of all three substrates to ensure potential induction of the respective hydrolases. Two fractions were then prepared for the test. For the first, a cell free extract was created by ultrasonic treatment of the cell pellet (see above). For the second, cells were centrifuged (8,000 rpm, 20 min). The supernatant was then passed through a 0.2 μm syringe filter and concentrated 10 times with 10 kDa Centriprep tubes (Merck). 30 μl of each fraction was applied into wells cut into a 1% agarose gel. The gel with wells was incubated for 3 days at 37°C. Proteolytic activity was detected by flooding the plate with 10% (w/v) trichloracetic acid. Amylolytic activity was detected by flooding with Lugol’s solution. Endo-glucanase activity was detected by Congo Red staining.

### Electron Microscopy

For electron microscopy, cells from 1 ml of pure culture grown with the *Staphylococcus* cells were harvested by centrifugation (8,000 rpm, 20 min), washed once, resuspended in 0.5 ml 1 M NaCl, pH 7 and stained for 1 min in 1% (w/v) uranyl acetate. The preparations were examined with a JEOL 100 model transmission electron microscope (Japan).

### 16S rRNA Gene Sequencing

For 16S rRNA gene sequencing, the cellular DNA was extracted and purified from 1 ml pure culture grown on peptone using the microbial DNA extraction kit (MoBio) according to the manufacturer’s instruction. The gene was amplified with 8f-1492r universal primer pair and the product sequenced by Sanger method using the same primers and two additional primers 530f and 1114f. The consensus sequence was obtained in BioEdit.

### Short Reads, DNA Extraction, and Sequencing

Genomic DNA was extracted from a freeze-dried pellet of the isolate using a modified version of the FastDNA SPIN Kit for Soil protocol (MP Biomedicals) as previously described (Costa et al., 2009), with a minor change being that the samples were processed in a bead beater twice for 30 s at setting 4.5 m/s.

Preparation of a genomic DNA library for short read sequencing was performed using the Nextera DNA Flex Library Prep protocol (Illumina), according to the manufacturer’s protocol. Shotgun metagenomic sequencing (2 × 300 bp) was performed using an Illumina MiSeq sequencer.

### Long Reads, DNA Extraction, and Sequencing

Genomic DNA was extracted from a freeze-dried pellet of the isolate using a modified version of the ‘Bacterial genomic DNA isolation using CTAB’ protocol (Version 3, Joint Genome Institute) designed to recover high molecular weight DNA. Wide bore tips were used throughout the protocol any time the DNA was handled directly.

The cell pellet was thawed and completely resuspended in 1x TE buffer at room temperature, treated with lysozyme (100 mg/mL) for 30 min at 37°C, then with 10% SDS and proteinase K (20 mg/mL) for 2 h at 55°C. Following cell lysis, 4.5 M NaCl and 10% CTAB solution (pre-heated to aid with viscosity) were added to assist in DNA purification with a 10 min incubation taking place at 65°C.

Organic extraction of DNA was performed using a 24:1 solution of chloroform:isoamyl and a 25:24:1 solution of phenol:chloroform:isoamyl. Organic extraction steps included: (1) chloroform:isoamyl treatment, (2) phenol:chloroform:isoamyl treatment, and (3) chloroform:isoamyl treatment. Following each step, the solution was mixed well by inversion (to minimize shearing) and centrifuged at high speed for 10 min. After each centrifugation step, the top layer (aqueous phase) was transferred to separate from the bottom layer (organic phase). The remaining aqueous phase was transferred to an empty tube along with 0.6 volumes of isopropanol and incubated at −20°C overnight to precipitate genomic DNA.

Following the overnight incubation, centrifugation occurred at 4°C for 15 min. The pelleted solution was rid of supernatant, washed with ice cold 70% ethanol to aid precipitation of the DNA, and allowed to dry at room temperature to remove residual supernatant. Once dried, the pellet was resuspended in 1x TE and treated with RNase A (10 mg/mL) at 37°C for 1 h to resolubilize the DNA and degrade any RNA. Following RNase treatment, 3 M Sodium Acetate (1/10 volume) and 100% ethanol (2.5 volumes) were added and the mixture was incubated at −80°C for 30 min. The DNA was then pelleted using centrifugation at 4°C for 20 min, washed with ice cold 70% ethanol, and allowed to dry at room temperature to remove residual supernatant. Once dried, the DNA was gently resuspended in DNase-free water, quantified using the Qubit dsDNA HS Assay Kit (Invitrogen, Thermo Fisher Scientific) and stored at 4°C prior to sequencing.

Preparation of a genomic DNA library for the isolate was performed using a modified version of the ‘Genomic DNA by Ligation (SQK-LSK109)’ protocol (Oxford Nanopore Technologies) designed for Flongle flow cells, including the addition/alteration of steps recommended by Tyson et al. (2018). Wide bore tips were used throughout the protocol any time the DNA was handled directly.

Prior to library preparation, genomic DNA was sheared using a 25-gauge needle and syringe (10 passes to yield desired length) then a clean-up step was performed with NGS beads (NucleoMag). Repair and end-prep of the DNA was performed following the manufacturers protocol with the only changes being to the volumes of DNA and reagents added (50 μL DNA sample, 7 μL NEBNext FFPE DNA Repair buffer, 3 μL NEBNext FFPE DNA Repair mix, 7 μL Ultra II End-prep reaction buffer, 3 μL Ultra II End-prep enzyme mix), incubation times (increased from 5 min to 30 min for both 20°C and 65°C incubations following reagent addition), and final eluate volume (increased from 60 μL to 67 μL). Adapter ligation and clean-up were performed following the manufacturers protocol with changes to the input DNA volume (increased from 60 μL to 66 μL), the amount of NEBNext Quick T4 DNA Ligase used (decreased from 10 μL to 4 μL), the incubation time following reagent addition (increased from 10 min to 60 min), and the final eluate volume (increased from 15 μL to 21 μL). As suggested in the manufacturers protocol, the DNA was quantified using the Qubit dsDNA HS Assay Kit (Invitrogen, Thermo Fisher Scientific) after each of the major steps identified above. Total quantity of DNA following each step was as follows: 27.38 μg post-needle shearing, 17.34 μg post-bead clean-up, 8.7 μg post-DNA repair and end-prep, and 4.5 μg post-adapter ligation and clean-up.

The DNA library was loaded onto the Flongle flow cell according to the manufacturer’s instructions. The sequencing run was then started using the MinKNOW program to track and visualize output and a MinIT to store the resulting data. The run progressed for ∼24 h and generated 0.38 GB of data, with an N50 value of ∼18,000.

### Genome Assembly, Annotation, and Comparative Genomics

For the Illumina short reads, BBDuk v38.06 (Bushnell, 2014) was used with options “qtrim = rl trimq = 15” to trim reads that contained adapter sequences, trim low quality ends, remove contaminants, and filter out reads shorter than 30 bp after clipping off the adapters.

For the Nanopore long reads, the raw fast5 files was base-called in real time on a MinIT using ONT’s Guppy v3.2.6 with the option “–config dna_r9.4.1_450bps_hac.cfg –qscore_filtering –min_qscore 7” and only reads passing initial quality checks during the run, which were placed into the “fastq_pass” folder, were used for downstream analysis. Porechop 0.2.4^1^ with default settings was used for adapter trimming. The adapter-trimmed reads were assembled using flye v2.7 (Kolmogorov et al., 2019) with “–nano-raw –genome-size 4m” options, leading to a single, circular contig. The contig was polished with adapter-trimmed nanopore sequences using BWA-MEM v0.7.17 (Li and Durbin, 2009) with the option “-x ont2d” and racon v1.4.3 (Vaser et al., 2017) with default parameters. The polishing process was repeated four times and was followed by one round of Medaka v0.11.5, a tool from Oxford Nanopore Technologies, using the r941_min_high_g303 model. Afterward, the Medaka-produced consensus contig was further polished using BWA-MEM and Pilon v1.23 (Walker et al., 2014) with the option “–fix all –changes” using the quality-controlled Illumina short reads. Finally, the Illumina-reads-polished circular contig was rearranged to start at the start position of the *dnaA* gene to produce the final draft genome. The draft genome was then annotated using MetaErg pipeline (Dong and Strous, 2019).

Amino acid and nucleotide fasta files for nine available *Wenzhouxiangella* genomes and twenty-three metagenome assembled genomes (MAGs) were downloaded from the NCBI assembly database (Table 1). Two genomes of the genus *Marinicella*, currently the sister-taxon to *Wenzhouxiangella* (Parks et al., 2018), were included for comparison. Average nucleotide identities were calculated with fastANI (Jain et al., 2018). Sets of orthologous genes were identified as follows: First, homology relations between proteins were calculated in an all-against-all NCBI blastp search (Camacho et al., 2008). Next, proteins were hierarchically clustered using mcl (for Markov clustering, Enright et al., 2002), at fineness 1.2, 1.4, 2, 4, and 6, according to the mcl manual. Each hierarchical cluster was parsed from course to fine and the subcluster with the highest # of species represented and the lowest # of species with multiple representative proteins was selected. If this cluster contained multiple representatives of a single species, the representative with the highest summed blastp score to all other representatives was designated as the ortholog, and the other proteins of that species were designated as paralogs. Phylogenetic relationships between *Wenzhouxiangella* whole genome sequences and MAGs were calculated as follows: First, relevant conserved single copy proteins were identified using hmmsearch with a set of hmm profiles used by gtdbtk (Parks et al., 2017, 2018). Next, sets of orthologs among the identified proteins were identified, as described above. Sets that lacked a representative in 25% of the species were discarded. One hundred and twenty-six remaining sets of orthologous proteins (listed in Supplementary Table 6 of Parks et al., 2017) were aligned using MAFFT (Katoh and Standley, 2013) with options “–maxiterate 1000 –localpair.” Poorly aligned regions were eliminated with gblocks with options “-t = p -b5 = h” (Talavera and Castresana, 2007) and all alignments were concatenated, leading to an alignment with 43,127 positions. From this, a bootstrapped maximum likelihood tree was created using RAxML, with options “-m PROTGAMMALG -f a -# 100,” as previously described (Hug et al., 2016). For 16S rRNA gene phylogeny, the model GTRGAMMA was used. Genes for production of secondary metabolites were identified with antiSMASH (Blin et al., 2019).

**TABLE 1:** Accession numbers and genome properties of *Wenzhouxiangella* and two *Marinicella* whole genome sequences and metagenome assembled genomes (MAGs).

			Genome properties					Checkm estimates	
Description	Accession #	Origin	Size (Mb)	% GC	N50 (kb)	#contigs	# 16S rRNA genes	Completeness	Contamination
*Wenzhouxiangella* sp. AB-CW3	CP061368	Kulunda soda lakes, Russia	3.84	61.8	3845	1	1	98.6	2.5
*Wenzhouxiangella marina*	GCF_001187785	Indian Ocean	3.68	65.3	3675	1	1	99.6	1.1
*Wenzhouxiangella* sp. XN201	GCF_011008905	Lake sediment, Shanxi, China	3.13	63.1	566	27	1	97.5	0.8
*Wenzhouxiangella limi (C33)*	GCF_010499265	Lake sediment, Shanxi, China	3.35	63.7	372	52	1	98.0	2.2
*Wenzhouxiangella* sp. W260	GCF_008725655	Sediment, Weihai, China	3.43	64.4	340	18	1	97.2	1.0
*Wenzhouxiangella* sp. XN24	GCF_011064545	Sediment, Shanxi, China	3.29	66.5	316	37	1	95.9	2.8
*Wenzhouxiangella sediminis*	GCF_003410055	Harbor sediment, China	3.51	65.4	136	54	1	97.8	1.2
*Wenzhouxiangella salilacus* sp. 15181	GCF_003410035	Lake water, Xinjiang, China	3.13	62.9	107	55	1	97.1	2.0
*Wenzhouxiangella* sp. 15190	GCF_003417465	Lake water, Xinjiang, China	3.13	62.9	107	55	1	97.1	2.0
*Wenzhouxiangella* sp. XN79A	GCF_012272825	Salt lake sediment, Shanxi, China	3.75	67.8	94	200	1	99.6	2.0
MAG	GCA_003567685	Soda lake sediment, Kulunda, Russia	2.74	61.7	55	107	0	97.6	2.1
MAG	GCA_003560975	Soda lake sediment, Kulunda, Russia	3.08	65.2	17	286	0	96.6	3.1
MAG Probe Lake 43	GCA_007695005	Soda Lake microbial mat, Cariboo, Canada	3.31	62.6	25	218	0	95.0	2.5
MAG	GCA_003565625	Soda lake sediment, Kulunda, Russia	2.81	63.6	63	95	0	94.9	3.1
MAG Deer Lake 32	GCA_007694055	Soda lake microbial mat, Cariboo, Canada	4.15	62.4	12	459	0	94.8	4.3
MAG Probe Lake 80	GCA_007694885	Soda lake microbial mat, Cariboo, Canada	2.60	65.0	21	215	0	91.9	3.9
MAG Hydrothermal Chimney 1	GCA_003233115	Hydrothermal Chimney Wall, Mid Atlantic Ridge	3.19	54.3	25	225	1	92.7	8.2
MAG Hydrothermal Chimney 2	GCA_003233095	Hydrothermal Chimney Wall, Mid Atlantic Ridge	2.89	55.4	18	232	0	92.3	2.5
*Marinicella sediminis*	GCF_002000055	Marine sediment, China	3.99	48.1	315	45	1	98.6	1.9
*Marinicella litoralis*	GCF_002591915	Seawater, Japan/Russian coast	3.35	42.4	857	36	1	97.8	1.9
MAG*	GCA_003564575	Soda lake sediment, Kulunda, Russia	3.18	60.3	22	215	0	82.1	15.0
MAG*	GCA_003556345	Soda lake sediment, Kulunda, Russia	3.42	60.4	18	277	0	80.8	12.0
MAG*	GCA_002722315	Pacific Ocean Water	3.53	64.1	923	53	0	80.7	2.5
MAG*	GCA_003558275	Soda lake sediment, Kulunda, Russia	2.85	61.7	6	520	0	76.0	5.6
MAG*	GCA_003556425	Soda lake sediment, Kulunda, Russia	2.70	62.9	10	346	0	75.4	5.1
MAG*	GCA_003556645	Soda lake sediment, Kulunda, Russia	2.62	60.3	12	308	0	73.5	2.6
MAG*	GCA_003561495	Soda lake sediment, Kulunda, Russia	2.55	60.2	13	257	0	72.9	1.7
MAG*	GCA_003557665	Soda lake sediment, Kulunda, Russia	3.34	63.2	14	345	0	70.8	6.9
MAG*	GCA_003562675	Soda lake sediment, Kulunda, Russia	2.43	61.9	12	308	0	67.2	0.0
MAG*	GCA_003556165	Soda lake sediment, Kulunda, Russia	1.95	62.6	15	176	0	66.4	1.8
MAG*	GCA_003557225	Soda lake sediment, Kulunda, Russia	2.17	64.2	6	398	0	62.5	5.8
MAG*	GCA_003568225	Soda lake sediment, Kulunda, Russia	4.41	59.2	8	681	0	61.8	5.3
MAG*	GCA_003562715	Soda lake sediment, Kulunda, Russia	1.82	62.6	7	293	0	56.8	1.2
MAG*	GCA_003568335	Soda lake sediment, Kulunda, Russia	1.43	61.9	5	295	0	51.0	1.7

Completeness and contamination were estimated using Checkm (Parks et al., 2015). *These Metagenome assembled genomes have not been analyzed further because of low estimated completeness and/or high contamination.

### Protein Extraction, Mass Spectrometry, and Proteomics Analysis

Protein was extracted in triplicate from three samples of the *Wenzhouxiangella* isolate grown on either *Staphylococcus*, casein, or peptone. Cell lysis was performed by mixing samples with SDT-lysis buffer (0.1 M DTT, 4% SDS) in a 10:1 ratio, and heating at 95°C for 10 min. Protein was then extracted from cell lysates using the filter aided sample preparation (FASP) protocol (Wisniewski et al., 2009). After protein extraction, an overnight digestion with 1.3 μg trypsin (Thermo Scientific Pierce, Rockford, IL, United States) in 50 mM ammonium bicarbonate at 37°C was conducted, followed by elution of peptides using 0.5 M NaCl.

Peptides were separated by an UltiMate^TM^ 3000 RSLCnano Liquid Chromatograph (Thermo Fisher Scientific, Waltham, MA, United States), and analyzed by a QExactive Plus hybrid quadrupole-Orbitrap mass spectrometer (Thermo Fisher Scientific) as previously described (Kleiner et al., 2017). In total, 400 ng of peptides were injected per sample, and a 120 min gradient was used for peptide separation.

A protein sequence database for the *Wenzhouxiangella* isolate was created using the predicted open reading frames from the circular genome. Sequences of common contaminating proteins were also added to this database^2^. Proteome Discoverer v 2.2x (Thermo Fischer Scientific) was used to analyze the MS/MS spectra. MS/MS spectra were searched against the protein sequence database using the Sequest HT node in Proteome Discoverer as described by Petersen et al. (2016). False discovery rates (FDRs) for peptide spectral matches (PSMs) and proteins were calculated and filtered using the Percolator Node and the FidoCT node in Proteome Discoverer, respectively (Spivak et al., 2009). Only peptides and proteins with an FDR below 5% were kept. Proteins with less than one unique peptide were discarded. Relative abundance of proteins was based on spectral counts and was calculated using normalized spectral abundance factors (NSAF) (Zybailov et al., 2006). A total of 1839 proteins were identified, accounting for 56% of the predicted proteome.

## Results and Discussion

### Enrichment and Isolation of a Pure Bacterial Culture Using *Staphylococcus* Cells as Growth Substrate

The enrichment medium was inoculated with a composite sample from hypersaline soda lake surface sediments. The medium contained 2 M total Na^+^ at pH 9.5. The culture started to show visual signs of *Staphylococcus* cell degradation after 2 weeks of incubation. After several 1:100 passages, visible increase of two dominant bacterial morphotypes was observed: straight thin rods in clusters, and long, twisted, filamentous cells. Plating of those onto solid medium with *Staphylococcus* cells produced colonies containing only the first phenotype. Follow up experiments indicated that the second morphotype was eliminated because the solid medium had two times lower salinity. The lower salinity still supported growth of the first morphotype, which had lower salt tolerance, but not of the second, apparently more salt-tolerant morphotype. The second morphotype was isolated into pure culture using liquid dilutions in medium containing 3 M total Na^+^ and was identified as a member of the genus *Natronospira*, within *Gammaproteobacteria*, formerly isolated from the same soda lakes with keratin as the substrate (Sorokin et al., 2017c). The first, less salt-tolerant bacterium formed flat “pan-cake” like colonies on 1 M Na^+^ *Staphylococcus* agar with a slight clearance of the opaque background right under the colonies. Several colonies were picked up under the binocular and inoculated into liquid medium at 2 M total Na^+^ with *Staphylococcus* cells. All colonies showed the ability to grow in these conditions and their cell morphology was identical, therefore a single culture was selected and designated strain AB-CW3.

**FIGURE 1:**
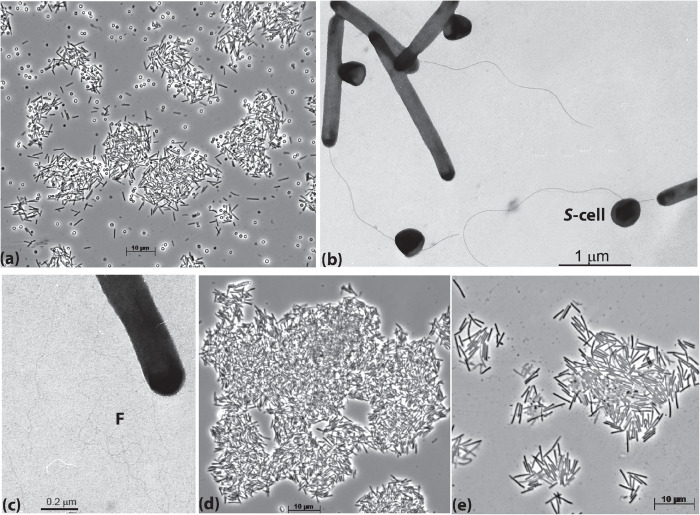
Cell morphology of strain AB-CW3 grown with *Staphylococcus* cells, shown as **S-cell (a–c)**, casein **(d)** and peptone/yeast extract **(e)**. Electron microscopy showed that cells of AB-CW3 often appeared attached to *Staphylococcus* cells and posses a single polar flagellum **(b)** and tufts of long thin fimbria-like filaments, shown as **F (c)**.

The cells of AB-CW3 grown with *Staphylococcus* cells in liquid culture were slim rods of variable length and formed large aggregates (Figure 1a). Electron microscopy showed that the AB-CW3 cells have a single, thick polar flagellum and often appeared to be attached to *Staphylococcus* cells (Figure 1b). In addition, they display tufts of very thin fimbria-like filaments, that might explain why they tend to aggregate to each other (Figure 1c). Aggregation of cells was also observed while growing with various proteins (Figure 1d) and peptones as the substrate. While growing with peptone, the cells were visibly larger, 4–5 μm × 0.33 μm, compared to 2.5–3 μm × 0.25 μm while growing on *Staphylococcus* (Figure 1e).

Comparison of the AB-CW3 16S rRNA gene to the NCBI database using blast showed that it is a member of the genus *Wenzhouxiangella*, part of the order *Xanthomonadales* within *Gammaproteobacteria*. *Wenzhouxiangella marina* is currently the only member of that genus with a complete 16S rRNA sequence, and AB-CW3 shared 96.7% identity with *W. marina.* The sequence identity with partial 16S rRNA gene sequences published for other *Wenzhouxiangella* genomes was up to 97% (Table 2), indicating that AB-CW3 may represent a new species of *Wenzhouxiangella.*

**TABLE 2:** Whole genome average nucleotide identity and 16S rRNA gene identity between strain AB-CW3 and other *Wenzhouxiangella* and *Marinicella*.

Target organism	% average nucleotide identity	% of genomes aligned*	% identity of 16S rRNA gene
AB-CW3	100.0	100.0	100.0
*Wenzhouxiangella salilacus* sp. 15181	78.8	29.2	97.2
*Wenzhouxiangella* sp. 15190	78.7	29.0	97.2
*Wenzhouxiangella* sp. XN201	78.7	29.9	97.0
*Wenzhouxiangella sediminis*	78.6	32.2	96.2
*Wenzhouxiangella limi (C33)*	78.2	27.5	95.6
MAG Deer Lake 32	78.0	24.7	–
*Wenzhouxiangella marina*	78.0	24.6	96.7
MAG Kulunda 2	78.0	24.4	–
MAG Kulunda 3	77.7	23.0	–
*Wenzhouxiangella* sp. XN79A	77.6	22.8	94.8
MAG Probe Lake 80	77.8	21.1	–
MAG Probe Lake 43	77.6	20.8	–
MAG Kulunda 1	77.6	20.0	–
*Wenzhouxiangella* sp. W260	77.1	10.0	91.0
*Wenzhouxiangella* sp. XN24	76.2	7.8	88.5
MAG Hydrothermal Chimney 1	Below detection limit		89.4
*Marinicella sediminis*	Below detection limit		87.5
*Marinicella litoralis*	Below detection limit		87.3

Note that all 16S sequences except for W. marina and AB-CW3 are only partially complete, so the reported % identity is an upper estimate of the actual % identity. No 16S gene was found in most metagenome assembled genomes (MAGs). *During computation of the average nucleotide identity, this is the percentage of the two whole genome sequences that actually align.

### Substrates and Growth Conditions of AB-CW3

**FIGURE 2:**
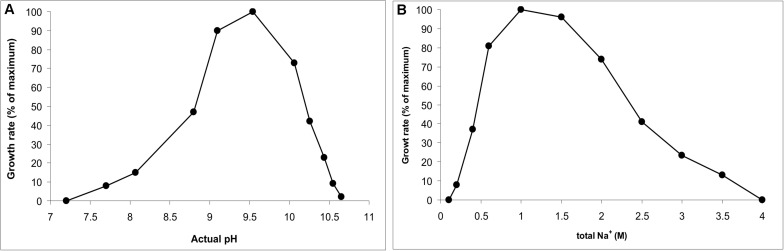
pH profile at 2 M total Na^+^ **(A)** and salt profile at pH 9.5 **(B)** for growth of AB-CW3 with peptone/yeast extract. Data represent mean values from duplicate parallel incubations at 37°C. The pH was measured at each sampling point.

AB-CW3 was enriched from hypersaline soda lakes, indicating that it might be a first haloalkaliphilic member of the genus *Wenzhouxiangella*. To confirm this, the salinity and pH profile for growth were measured with peptone and yeast extract as substrates. The pH profile was measured at 2 M total Na^+^ and the salt profile at pH 9.5, as those were the conditions at which the strain was enriched and isolated. The results (Figure 2) showed that AB-CW3 is an obligate alkaliphile, with a pH range for growth between 8 and 10.5 (optimum at pH 9.5). In the sodium carbonate buffer, it grew at a broad range of salinity, between 0.2 and 3.5 M total Na^+^. As growth was optimal between 0.6 and 1.5 M Na^+^, AB-CW qualifies as a moderately salt-tolerant alkaliphile.

The most interesting trait of AB-CW3 was the ability to use bacterial cells as an energy and carbon source. It was enriched and isolated with cells of *Staphylococcus aureus*. To investigate whether it was also able to grow with other Gram-positive bacteria, AB-CW3 was incubated with cells of *Micrococcus* and *Isoptericola*. The latter is a halo-alkaliphilic Actinobacterium isolated from a saline soda soil in the same area as AB-CW3 (Sorokin et al., 2017b). Both *Micrococcus* and *Isoptericola* supported growth of AB-CW3. While growing with all these bacteria as the substrate, visible growth was detectable after 3 days or later, while massive growth with substantial degradation of the prey bacteria occurred after 10–14 days. In control experiments without addition of AB-CW3 cells, no degradation of prey bacteria was observed and the optical density remained constant.

Degradation of prey bacteria was never complete, even after heat-treatment. Incomplete degradation was apparent by the formation of much less refractile “ghosts” of *Staphylococcus* cells which remained behind in the medium. This indicated that AB-CW3 was unable to consume all parts of its prey. Based on its ability to consume proteins and peptones, it likely consumed cellular proteins, but might not be able to consume other components, such as polysaccharides, lipids, murein and DNA. This was confirmed by testing various growth substrates at pH 9.5 and 2 M total Na^+^. Growth was observed on soluble proteins, such as bovine serum albumin (BSA), gelatine and hemoglobin. Growth was also observed for insoluble proteins, such as casein, lactalbumin, collagen and powdered alpha-keratin. AB-CW3 grew fastest with casein and degradation was incomplete for alpha-keratin. Yeast extract and various peptones also promoted fast growth. Carbohydrates (18 different sugars and starch) did not support growth, except maltose and dextrin, which slightly stimulated growth in the presence of a small amount of peptone.

When grown with casein, AB-CW3 cells displayed alkaline proteinase enzyme activity. This activity was associated with the AB-CW3 cells themselves, and was not detectable in the culture supernatant. As the casein would need to be hydrolyzed before it can be imported into cells, we would expect the alkaline proteinases to be located at the cell surface. Alpha-1,4 and beta-1,4 endoglucanase activities were not observed. Lipase activity was also missing, as shown by absence of growth with olive oil. Together, these results showed that AB-CW3 is a dedicated proteolytic bacterium that needs to make direct cell contact (see also Figure 1b) with proteinaceous substrates, including living cells.

### Phylogeny and Comparative Genomics

Using a combination of long and short read sequencing, the circular genome of AB-CW3 was fully resolved. It consisted of 3,844,697 nucleotides at 61.8% GC and featured a single rRNA operon (16S-tRNA-tRNA-23S-5S), 46 transfer RNA genes and 3,271 predicted coding sequences, at a coding density of 91%. For comparison, 17 currently available *Wenzhouxiangella* genomes, from sequenced isolates or binned from metagenomes (Table 1) were 2.6–4.2 Mb large at 61–68% GC. Use of short-read Illumina sequencing yielded fragmented genomes (consisting of 18–509 contigs) for all existing species except *W. marina*. Now, using nanopore sequencing, AB-CW3’s genome became the second closed, complete and circular *Wenzhouxiangella* genome.

**FIGURE 3:**
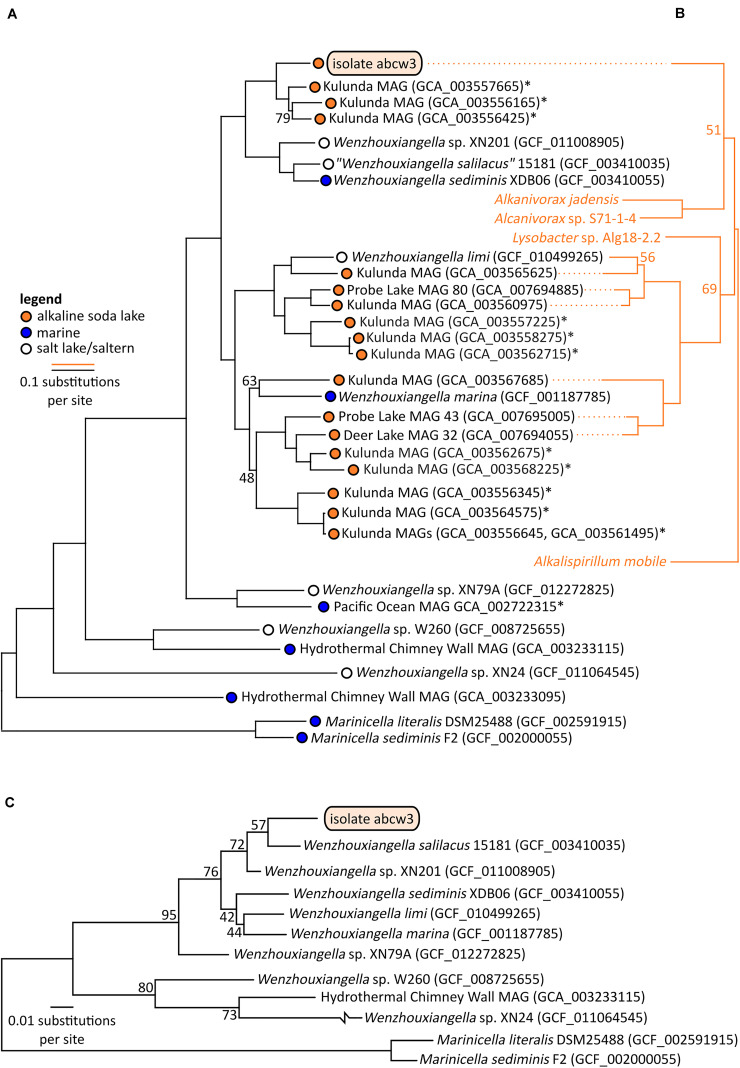
Bootstrapped maximum likelihood phylogenetic trees of *Wenzhouxiangella* and *Marinicella* isolates and metagenome assembled genomes (MAGs) based on a concatenated alignment of 126 conserved single copy genes (43,127 positions) (Parks et al., 2017) **(A)**, and sodium-pumping NADH:ubiquinone oxidoreductase subunits NqrABC (1,098 positions) **(B)**, and based on 16S rRNA genes **(C)**. *indicate incomplete metagenome assembled genomes (<90% completeness, as estimated by CheckM), which generally do not include 16S rRNA genes. Bootstrap values are indicated at nodes when lower than 99%.

An established set of single copy conserved genes, as well as 16S rRNA genes, were used to compute phylogenetic relationships among members of *Wenzhouxiangella* (Figure 3). Two species of *Marinicella* were used as the outgroup. The genus *Marinicella* is, currently, most closely related to *Wenzhouxiangella*, according to GTDB (Parks et al., 2018). This relationship is still quite distant, as both genera are in separate families, *Wenzhouxiangellaceae* and *Marinicellaceae* respectively, both within the order *Xanthomonadales*. Note that according to the NCBI taxonomy, *Wenzhouxiangella* is part of the order *Chromatiales*, whereas *Marinicella* is part of the order *Oceanospirillales*. Based on the bootstrap values of Figure 3, GTDB’s marker gene approach led to a much more robust phylogeny of *Wenzhouxiangellaceae* than the classical 16S rRNA gene based approach, but a complete re-assessment of the phylogeny of *Gammaproteobacteria* was beyond the scope of our study. Regardless of the taxonomic relationships, as a marine, aerobic, peptide-degrading gammaproteobacterium (Romanenko et al., 2010), *Marincella* was a meaningful comparator organism.

Within *Wenzhouxiangella*, bacteria from alkaline soda lakes formed a single clade, together with species isolated from marine environments and hypersaline habitats with neutral pH (Figure 3).

Average nucleotide identities shared between any of these genomes were below 80% (Table 2). According to current views on the definition of microbial species, when two genomes have more than 95% average nucleotide identity, they can be considered the same species. Two different species have less than 83% sequence identity (Jain et al., 2018). Thus, AB-CW3 represents a new species within the genus *Wenzhouxiangella*, for which we propose the provisional name “*Wenzhouxiangella alkaliphila*.” For this name to become taxonomically valid, additional phenotypical characterization will be necessary.

Orthologous genes shared between multiple *Wenzhouxiangella* species were compared to identify core and accessory genes, shared and differential gene content among the genomes (Supplementary Data). Most genes of AB-CW3 (2,003 genes, 61%) were orthologous to a gene in another *Wenzhouxiangella* species. About half of those were also present in *Marinicella* genomes.

This core genome of 1,126 genes, present in both *Marinicella* and ten or more *Wenzhouxiangella* genomes, defined shared metabolic and conserved features. Apart from genes involved in central metabolism, the central dogma and biogenesis of a Gram-negative cell envelope, the core genome featured genes for respiratory complexes I-V, enabling aerobic respiration. Three different terminal oxidase complexes were present, including high affinity *cbb3* cytochrome *c* oxidase and *bd* quinol oxidase. This indicated a universally aerobic lifestyle among members of these families and adaptation to a range of oxygen concentrations. The core genome also included a type II protein secretion system and the oligopeptide transporter *opp*.

Among 238 genes present in ten or more *Wenzhouxiangella* genomes and missing in *Marinicella*, the presence of lactate dehydrogenase indicated a potential for basic fermentation in *Wenzhouxiangella*, but not in *Marinicella*. In addition, squalene synthase and the mevalonate pathway for isoprenoid biosynthesis were specific to *Wenzhouxiangella.* Squalene and other isoprenoids make up as much as 50% of total membrane lipids in halo-alkaliphiles, and have proposed to function as proton-permeability barriers in membranes of these bacteria (Haines, 2001; Hauß et al., 2002). Thus, retention or acquisition of those genes by the ancestor of *Wenzhouxiangella* might have been essential for the later transition of some *Wenzhouxiangella* bacteria to alkaline soda lake environments. Finally, *Wenzhouxiangella* genomes encoded the *mla* system for glycerophospholipid transfer across the periplasm, and genes similar to *mtrAB*, part of an electron transport system known from iron reducing bacteria, which may in this case be involved in iron uptake.

A comprehensive study of gene losses and acquisitions among *Xanthomonadales* bacteria may shed more light on the role of habitat transitions in the emergence of the extant genera.

Only 19 orthologs were clearly specific to growth in halo-alkaliphilic environments. These genes were found in all six complete or nearly complete genomes of halo-alkaliphilic *Wenzhouxiangella*, but were absent in at least eight out of nine species from the pH-neutral environments. All of these genes encoded for membrane complexes, periplasmic and outer membrane proteins. The sodium dependent NADH:ubiquinone oxidoreductase complex (NqrA-E) was among this inventory, as well as two genes encoding outer membrane porins. The remainder were genes with unknown functions. Phylogenetic analysis of subunits NqrABC of the NADH:ubiquinone oxidoreductase complex showed that the two major clades of alkaliphilic *Wenzhouxiangella* may have acquired this complex from different hosts (Figure 3). Even though the Nqr complex is also common among marine bacteria, *W. marina* apparently lost its Nqr complex after transitioning back from alkaline soda lake to the marine environment. A relative recent transition of this species to the marine environment is consistent with the acquisition of multiple genomic islands by lateral gene transfer from other marine bacteria (Lee et al., 2015).

Consistent with the microscopic observations, AB-CW3 displayed a complete set of flagellar and fimbrial biosynthesis genes. The flagellum is a rare feature among *Wenzhouxiangella* and *Marinicella*, because in our dataset, only *Wenzhouxiangella limi* shared the potential for this type of bacterial mobility. AB-CW3 and *W. limi* flagellar genes were very distantly related to those found in the gammaproteobacterium *Chromatocurvus*, providing no hints of a traceable lateral gene transfer event.

Finally, unique among *Wenzhouxiangella*, AB-CW3 possessed genes for uptake and biosynthesis of the osmolyte ectoine. Osmolytes are an essential adaptation to marine and soda lake environments, but apparently the repertoire of osmolytes used is not fixed among members of this genus.

### Denitrification

Among *Wenzhouxiangella* genomes, only AB-CW3 encoded and expressed genes for a periplasmic (Nap) nitrate reductase, cytochrome *cd*_1_ nitrite reductase NirS and a clade-I, twin-arginine-dependent nitrous oxide reductase NosZ. Although no gene had high similarity to known nitric oxide reductases, two heme-copper-oxidase-family enzymes were present (in addition to the two already mentioned cytochrome c oxidase complexes). These could in theory function as nitric oxide reductases.

**FIGURE 4:**
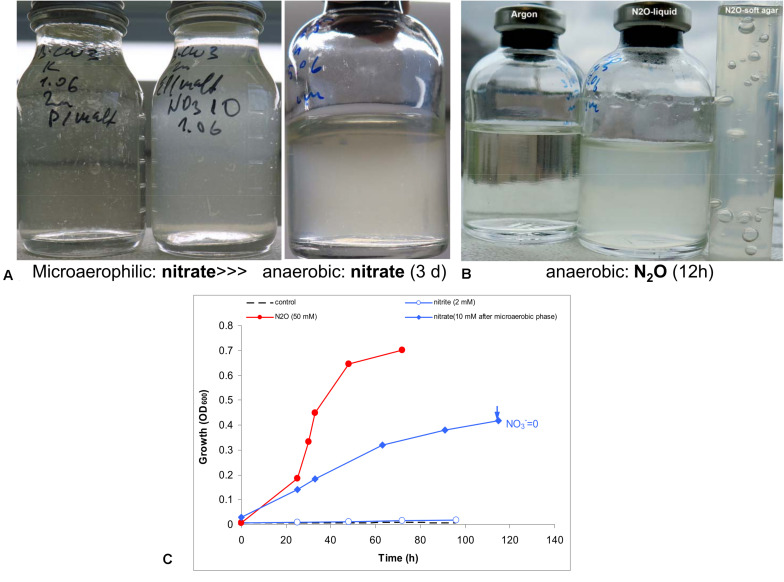
Evidence for anaerobic growth of AB-CW3 by denitrification. **(A)** Gradual adaptation to anaerobic growth with nitrate (the third flask) after micro-aerophilic growth (the second flask); the direct transfer from an aerobic culture to anoxic conditions did not result in growth with nitrate (the first flask). **(B)** Anaerobic growth with nitrous oxide was rapid and effective. **(C)** Anaerobic growth occurred with nitrate and nitrous oxide, but not with nitrite. However, no nitrite accumulated during nitrate reduction. The data are mean values from duplicate experiments.

Anaerobic growth with nitrate was tested experimentally with peptone and maltose as the carbon and energy source. Initial test produced negative results. However, when the culture was first cultivated statically in a closed flask with 30 ml culture and only 3 ml air, a clear difference was observed between the control and the culture supplemented with nitrate. The latter then was transferred into fully anoxic conditions and showed a steady anaerobic growth with complete nitrate consumption (Figure 4). A second transfer from this culture already directly supported a slow anaerobic growth with nitrate with a doubling time of 25 h. This approach did not work with nitrite, even at low concentration (2 mM). The most active anaerobic growth was observed with nitrous oxide as the electron acceptor (doubling time 6 h), and active formation of N_2_ was visible in the soft agar cultures, after incubation with nitrous oxide (Figure 4).

In combination, genomic and experimental evidence indicated that AB-CW3 effectively reduces nitrate and especially nitrous oxide. Nitrous oxide reductase was also among the most highly expressed genes in all three proteomes. Although it did reduce nitrite to nitrous oxide, these conversions occurred at a much lower rate. Presence and activity of both nitrate reductase and nitrous oxide reductase, while nitrite and nitric oxide reductases are lacking or less effective, is common among bacteria (e.g., Chen et al., 2017). Although nitrate reduction has been reported before (Zhang et al., 2020) among *Wenzhouxiangella*, nitrous oxide reduction and complete denitrification are unique traits of AB-CW3 so far.

### Proteomics and Proteolytic Lifestyle

Using proteomics, expression was demonstrated for 1,827 genes with cells grown aerobically on three different substrates: peptone, casein and killed *Staphylococcus* cells (Supplementary Data). Differences in proteomes of cells grown on these different substrates were minor, indicating that AB-CW3 constitutively expresses a set of genes for proteolysis and peptide import, a set that enables it to feed on a range of different proteinaceous substrates.

The most abundant protein of AB-CW3 was a TonB-dependent outer membrane receptor. The TonB complex consists of subunits TonB, ExbB and ExbD, anchored in the cytoplasmic membrane and spanning the periplasm. The complex uses the proton motive force to drive active import across the outer membrane, via TonB-dependent receptors. These receptors are best known for their role in uptake of siderophores and antimicrobial peptides, such as colicin. They are very common among Gram-negative bacteria. For example, *Caulobacter crescentus* produces 65 of these receptors (Noinaj et al., 2010). AB-CW3 expressed all three subunits of the TonB complex as well as fourteen out of sixteen TonB-dependent receptors encoded in its genome. Out of sixteen receptors, only four were orthologous to receptors found in other *Wenzhouxiangella* genomes. All *Wenzhouxiangella* and *Marinicella* genomes contained between 10 and 42 of these receptor genes, and most of those were unique in our dataset. Three receptors were among the ten most highly expressed genes of AB-CW3, and two of those were not present in any other *Wenzhouxiangella* genome.

As AB-CW3 was growing on peptides, which need to be transported across the outer membrane, and TonB-dependent receptors are known to import peptides, these highly expressed receptors were likely responsible for peptide or protein uptake. In addition to these receptors, AB-CW3 expressed 37 out of 45 proteases and peptidases with a signal peptide, indicating their translocation across the cytoplasmic membrane. This set did not include known housekeeping enzymes, such as chaperones and signal peptidases, which were obviously not involved in peptide or protein uptake.

About half of those were shared with other *Wenzhouxiangella.* Among peptidases, a gluzincin metallopeptidase similar to the periplasmic *E. coli* Oligopeptidase A was most highly expressed. We were unable to infer whether some of those peptidases were also translocated across or inserted into the outer membrane for display on the cell surface. For this, *Wenzhouxiangella* might use their type II secretion system. But taking into account, that no proteolytic activity was found to be excreted into culture medium in AB-CW3, it is safe to assume that the acting extracellular lytic proteases are anchored at the outer membrane in this bacterium.

A comprehensive search for additional secretion systems, production of secondary metabolites and known toxins, revealed a gene cluster containing the type I secretion system genes *hlyBD* (with *tolC* encoded elsewhere in the genome) next to a cluster with genes similar to lantibiotic biosynthesis proteins *lanAM*. Lantibiotics (Lanthipeptides) are post-translationally modified antimicrobial peptides produced by, and targeting, Gram-positive bacteria like *Staphylococcus* (Van Kraaij et al., 1999). Their production requires *lanAM*, with *lanA* encoding the peptide precursor, and *lanM* a dehydrogenases/thioesterase that post-translationally crosslinks the precursor’s amino acids with thioester bridges. Cleavage of a N-terminal leader sequence constitutes the final step in the biosynthesis of these antibiotics. Based on AB-CW3’s *lanA* gene (abcw3| 00290), AntiSMASH predicted a class II lantibiotic with the sequence **C**GGMGG**C**QPNPF**Dha**A**Dhb**L**C**PDG**Dhb**WYQ**C**P, with crosslinked cysteine residues shown in bold, **Dha**, Didehydroalanine and **Dhb**, Didehydrobutyrine. Expression of *lanA* was not detected, as would be expected for a heavily modified peptide. However, expression of *lanM*, immediately downstream of *lanA*, was four times higher in the presence, than in the absence of *Staphylococcus* cells. AB-CW3 *lanM* was most similar to a gene found in *Melittangium boletus* (Treuner-Lange et al., 2017), affiliated with *Myxococcales*, a clade that also includes known predatory bacteria (Mu et al., 2020). No other *Wenzhouxiangella* encoded a similar gene. Leader peptide cleavage could be performed by periplasmic peptidase abcw3| 00280, immediately upstream of the type I secretion system genes. This type of antibiotics has so far only been studied for Gram-positive bacteria, but the associated genes are sometimes present in genomes of Gram-negative bacteria, like AB-CW3. The target and method of action of the new Lanthipeptide is still unknown, but known Lantibiotics lead to cell death, for example by compromising the integrity of the cytoplasmic membrane (Van Kraaij et al., 1999). Based on AB-CW3’s ability to prey on *S. aureus* and other Gram-positive bacteria, and lack of apparent enzymes for murein degradation, the action of the Lantibiotic may ultimately lead to the partial disintegration of the target cells. This will merit future experimental work, which could lead to discovery of new antibiotics. Compared to other predatory bacteria, AB-CW3 is a facultative predator, based on its mechanism of action by production of an antibiotic, its apparently broad host range, its ability to grow in the absence of its prey and relatively complete central metabolism (Mu et al., 2020).

## Conclusion

Within the Order *Xanthomonadales* in *Gammaproteobacteria*, the sister genera *Wenzhouxiangella* and *Marinicella* form a clade of generally non-motile, marine, proteolytic, aerobic bacteria. Unlike *Marinicella, Wenzhouxiangella* produce squalene, a neutral membrane lipid, important for the Na^+^/H^+^ impermeability of the cell membrane in extremophilic prokaryotes. *Wenzhouxiangella* might have transited from the pH-neutral saline habitats to alkaline soda lakes multiple times. Acquisition of about 20 genes, including those encoding the sodium-dependent form of the respiratory complex I, was needed to make the transition. In addition, *Wenzhouxiangella* strain AB-CW3 possesses flagella and fimbria and performs denitrification, rare traits among members of the genus. Analysis of its circular genomic chromosome sequence combined with comparative proteomics, indicated that TonB-dependent receptors play a key role in protein uptake of this dedicated proteolytic bacterium. All *Wenzhouxiangella* genomes encoded 10–42 mostly genus-specific TonB receptors, providing each species with a unique niche in terms of its protein substrates. Unique among *Wenzhouxiangella*, AB-CW3 expressed genes for production of a class II lantibiotic, targeting Gram-positive bacteria, consistent with an experimentally validated ability to grow on cells of *Staphylococcus* and two other Gram-positive bacteria. As strain AB-CW3 is only distantly related to other members of the genus, we propose to provisionally name it “*Wenzhouxiangella alkaliphila*.”

## Data Availability Statement

The DNA sequence data is available at the NCBI, accession numbers BioProject PRJNA657903, BioSample SAMN15851626, Short Read Archive SRX8967578 and SRX8967579. The whole genome sequence is available at the NCBI with accession number CP061368. The proteomes are available at PRIDE, accession number PXD021036.

## Author Contributions

DS isolated the bacterium and performed physiology experiments. DM implemented and performed nanopore and Illumina sequencing. JZ performed proteomics and proteomics data processing, interpretation. XD implemented and performed assembly of nanopore and Illumina reads and performed annotation. MS performed phylogeny and comparative genomics. MS and DS wrote the manuscript with input from all other co-authors. All authors contributed to the article and approved the submitted version.

## Conflict of Interest

The authors declare that the research was conducted in the absence of any commercial or financial relationships that could be construed as a potential conflict of interest.

## Footnotes

1 https://github.com/rrwick/Porechop

2 http://www.thegpm.org/crap/
